# Concordance between administrative health data and medical records for diabetes status in coronary heart disease patients: a retrospective linked data study

**DOI:** 10.1186/1471-2288-13-121

**Published:** 2013-10-01

**Authors:** Lee Nedkoff, Matthew Knuiman, Joseph Hung, Frank M Sanfilippo, Judith M Katzenellenbogen, Tom G Briffa

**Affiliations:** 1 School of Population Health, The University of Western Australia, 35 Stirling Highway, Crawley, WA 6009, Australia; 2 School of Medicine and Pharmacology, Sir Charles Gairdner Hospital Unit, The University of Western Australia, Crawley, WA, Australia; 3 Combined Universities Centre for Rural Health, The University of Western Australia, Crawley, WA, Australia

**Keywords:** Coronary heart disease, Diabetes, Administrative data, Hospital morbidity data, Concordance, Comorbidity

## Abstract

**Background:**

Administrative data are a valuable source of estimates of diabetes prevalence for groups such as coronary heart disease (CHD) patients. The primary aim of this study was to measure concordance between medical records and linked administrative health data for recording diabetes in CHD patients, and to assess temporal differences in concordance. Secondary aims were to determine the optimal lookback period for identifying diabetes in this patient group, whether concordance differed for Indigenous people, and to identify predictors of false positives and negatives in administrative data.

**Methods:**

A population representative sample of 3943 CHD patients hospitalized in Western Australia in 1998 and 2002–04 were selected, and designated according to the International Classification of Diseases (ICD) version in use at the time (ICD-9 and ICD-10 respectively). Crude prevalence and concordance were compared for the two samples. Concordance measures were estimated from administrative data comparing diabetes status recorded on the selected CHD admission (‘index admission’) and on any hospitalization in the previous 1, 2, 5, 10 or 15 years, against hospital medical records. Potential modifiers of agreement were determined using chi-square tests and multivariable logistic regression models.

**Results:**

Identification of diabetes on the index CHD admission was underestimated more in the ICD-10 than ICD-9 sample (sensitivity 81.5% versus 91.1%, underestimation 15.1% versus 4.4% respectively). Sensitivity increased to 89.6% in the ICD-10 period using at least 10 years of hospitalization history. Sensitivity was higher and specificity lower in Indigenous patients, and followed a similar pattern of improving concordance with increasing lookback period. Characteristics associated with false negatives for diabetes on the index CHD hospital admission were elective admission, in-hospital death, principal diagnosis, and in the ICD-10 period only, fewer recorded comorbidities.

**Conclusions:**

The accuracy of identifying diabetes status in CHD patients is improved in linked administrative health data by using at least 10 years of hospitalization history. Use of this method would reduce bias when measuring temporal trends in diabetes prevalence in this patient group. Concordance measures are as reliable in Indigenous as non-Indigenous patients.

## Background

Linked administrative health data provide a unique resource for investigating whole-population diabetes mellitus (‘diabetes’) prevalence in different patient groups. Administrative data systems are commonly designed to collect resource utilisation data rather than as repositories for research purposes, with recording of comorbid conditions often not required at every hospital admission [1]. An understanding of the reliability of hospital data can assist in accurately estimating the impact of diabetes in the high-risk coronary heart disease (CHD) patient population [2,3]. In unlinked administrative databases where comorbidity information is obtained from a single admission, it is important to understand the reliability of coding and which patient groups may be under or overestimated from this data source. Use of information from a single admission only could underestimate diabetes prevalence and inaccurately identify diabetic patients. Applying a lookback period to identify prior admissions in which diabetes was recorded can increase detection of diabetes status in linked datasets [4]. Many studies identify diabetes using a lookback of less than two years [5,6] but information is limited on the optimal length of hospitalization history required and whether this method is consistent over time.

Changes in International Classification of Disease (ICD) versions could potentially impact the recording of conditions such as diabetes. Significant changes in coding directives for diabetes were introduced in Australia in 2000 [7], with a subsequent 20% increase in the number of diabetes-related admissions to 2003–04 [8]. Accordingly, the national health statistics body in Australia does not measure trends spanning the ICD-9 and ICD-10 periods for overall diabetes-related hospitalizations [8]. Whether these coding changes have impacted in the same manner on CHD hospitalizations is unknown.

There is limited available data on the accuracy of recording of diabetes in population sub-groups, including in Indigenous Australians. The risk of diabetes in Indigenous people is known to be many times higher than in the general population [9], and because a high proportion of Indigenous people reside in rural and remote areas, they are more likely to be admitted to a non-metropolitan hospital. These factors could impact on identification of diabetes from administrative data for these patients. With high and increasing incidence of diabetes in this group [9,10] it is imperative that the utility of administrative data for identifying diabetes status is investigated.

The primary aim of this study was to measure the concordance of administrative data and medical records for the recording of diabetes in a sample of CHD patients, and determine whether this has changed over time. Secondary aims were to determine the optimal lookback period for identifying diabetes in this patient group, whether concordance differed for Indigenous people with CHD, and to identify predictors of false negatives and false positives in administrative data.

## Methods

### Study setting

The current study was performed in the state of Western Australia (WA) which is representative of the major sociodemographic and health economic indicators for Australia [11]. The population of WA in 2004, the latter period of the study, was 1.99 million, with 75% residing in the capital city, Perth [12]. Indigenous people comprise 3.5% of the WA population [13], with around 65% living in regional, rural or remote areas [14]. Data was sourced from the population-based electronic linked health database (WA Data Linkage System) which is managed by the Department of Health WA and has been used extensively for health-related research [15]. The current study used two of the system’s core databases - the Hospital Morbidity Data Collection (HMDC) and the Mortality register. Statutory requirements mean that all hospitalizations and deaths in WA are recorded within these collections. The datasets are linked by probabilistic matching based on name, date of birth, gender and address, with manual clerical checking of uncertain links, and are regularly audited for quality [16]. Hospital discharge diagnoses are coded in the HMDC by trained coders using the prevailing ICD version and relevant modifications (ICD-9 from 1978, and ICD-10 from July 1, 1999).

### Study sample

The study sample was selected from two existing projects: Monitoring CHD in the Modern Era (Study 1), and More Informed Action to Improve Aboriginal Heart Health in WA (Study 2). The sampling frames for these studies have been described elsewhere [17]. A stratified sample of patients aged 35–79 years with a hospital discharge diagnosis of any cardiac condition or chest pain in 1998 or 2003, admitted to a major public or private metropolitan hospital, was identified from a linked dataset containing all cardiovascular (CVD) morbidity and mortality records. The second study similarly identified all Indigenous patients and a sample of non-Indigenous patients, aged 25–79 years, admitted to any metropolitan or rural hospitals in 2002–04. Hospital record review for these samples was undertaken and information collected and stored in a medical records database. Because of the overlap in time period between the 2003 sample in Study 1 and the Study 2 sample (2002–04), 134 patients appeared in both sampling frames. These were included only once in the medical records database.

Patients in the medical records database were included in the current study if they had a principal discharge diagnosis of CHD recorded in the HMDC (ICD-9-CM 410–414, ICD-10-AM I20-I25), because of the high recording accuracy of CHD in the principal compared with secondary diagnosis fields [18]. The first CHD admission for each patient in each time period was defined as the ‘index admission’ and selected for inclusion in the study. The administrative data for the CHD patients were linked to the medical records database via a unique identification number assigned to every hospital admission in WA. Because of known underestimation of identification of Indigenous status in hospital discharge data [19], a patient was included as Indigenous if 25% or more of all of their HMDC records since 1980 were recorded as Indigenous.

### Medical record review

Trained research assistants collected data from medical records. Thirty-nine admissions could not be reviewed due to missing medical notes. Data were obtained from admission notes from the emergency department and inpatient medical records, and each comorbidity documented as present, absent, or not recorded. Treatment of diabetes with insulin or oral hypoglycaemic drugs was identified from inpatient and discharge drug records for the admission under investigation. Patients were classified as having diabetes if it was documented as ‘present’ in the medical notes or if drug treatment for diabetes was identified. Patients with ‘not recorded’ as their diabetes status and no diabetic drugs recorded (n = 66) were classified in the no diabetes group, and a sensitivity analysis with these patients removed showed minimal difference in all concordance measures across the two samples.

Data quality was initially assessed by review of three medical records by all research assistants within two weeks of commencing data collection. Medical records of a total of 11 patients were subsequently assessed by all data collectors in each study. The observed agreement between data collectors in Study 1 was high for selected medical history (92%) and drugs (100%) and similarly for Study 2 (93% and 87% respectively).

### Identification of diabetes status from hospital discharge data

ICD-9-CM was in use in WA at the time of the 1998 admissions, and ICD-10-AM at the time of the 2002–04 sample. Diabetes (Type 1, Type 2, other specified or unspecified diabetes mellitus) was identified in hospital discharge data for the CHD sample if coded in any of 21 diagnosis fields (ICD-9/ICD-9-CM 250, ICD-10-AM E10-E14), using a range of lookback periods for each individual patient – index CHD admission only, and 1, 2, 5, 10 and 15 years prior to the CHD admission.

Approval for this study was obtained from the Ethics Committees of The University of Western Australia and the Department of Health WA, and from the Western Australian Aboriginal Health Ethics Committee.

### Statistical analysis

The crude prevalence of diabetes in the CHD patient sample was calculated for the index admission and each lookback period using the administrative data, and from the medical records database. Observed agreement, sensitivity, specificity, positive predictive value (PPV), negative predictive value (NPV) and Kappa were used as measures of concordance between administrative and medical records data, with the latter designated the reference standard. Concordance measures were calculated for each lookback period and also for sub-group and supplementary analyses. The percentage of under or overestimation of diabetes recording in hospital discharge data was calculated by (Sensitivity/PPV - 1) ×100 [17]. All analyses were stratified by period, with the 1998 sample corresponding to ICD-9, and the 2002–04 sample to ICD-10. Differences in prevalence between medical records and administrative data were tested using McNemar’s test, and concordance measures between the two ICD samples were tested using a Pearson chi-square or z-test (for under/overestimation).

Because of the possible impact of sociodemographic and clinical factors on concordance, and the potential for changing criteria for admission to hospital for CHD, variables derived from the administrative data on the index CHD admission were examined for their association with false positives and false negatives, separately for the ICD-9 and ICD-10 samples. Univariable associations were analysed using the Pearson chi-square test (or Fisher’s exact test where cell counts were small). The variables tested were length of stay (1–2, 3–5, ≥6 days), age group (25–50, 51–65, 66–79 years), gender, admission type (elective versus emergency), Indigenous status, Charlson Comorbidity Index (excluding diabetes; 0, 1–4, ≥5), number of comorbidities on the index admission (excluding diabetes; 0–3, 4–7, ≥8), hospitalization in the previous 90 days, hospital location (metropolitan versus rural), hospital type (public versus private), transfer in or out on index CHD admission, in-hospital death, principal diagnosis (myocardial infarction, unstable angina, other CHD), and first-ever versus recurrent CHD admission. First-ever CHD admission was identified where there was no CHD admission in the previous 15 years. Variables were tested using binary values unless otherwise indicated and all analyses undertaken separately for each ICD period. Significant univariable variables in each period were entered into a multivariable logistic regression model and odds ratios with 95% confidence intervals (CI) were calculated. All data analyses were undertaken using SAS (version 9.3, Cary, NC, USA) and statistical significance for all analyses was set at p < 0.05.

## Results

The final study sample comprised 3943 index CHD admissions, with 24 patients having an admission in both periods. Table 1 shows the clinical and demographic characteristics of the sample. There were 1685 patients in the ICD-9 sample, and 2258 in the ICD-10 sample, with Indigenous patients comprising 23.2% of the latter sample. The majority of cases (94.1%) were admitted for acute coronary syndromes (myocardial infarction or unstable angina).

**Table 1:** Characteristics of the study sample

	**ICD-9**	**ICD-10**	**Whole sample**
	**(n = 1685)**	**(n = 2258)**	**(n = 3943)**
Mean age, years (SD)	64.1 (10.8)	61.1 (12.3)	62.4 (11.8)
Males	1154 (68.5)	1530 (67.8)	2684 (68.1)
Indigenous people	25 (1.5)	525 (23.2)	550 (13.9)
Principal diagnosis			
MI	860 (51.0)	1061 (47.0)	1921 (48.7)
UA	737 (43.7)	1052 (46.6)	1789 (45.4)
Other CHD	88 (5.2)	145 (6.4)	233 (5.9)
Length of stay, days			
1-2	284 (16.8)	635 (28.1)	919 (23.3)
3-5	714 (42.4)	1011 (44.8)	1725 (43.8)
≥6	687 (40.8)	612 (27.1)	1299 (32.9)
Charlson index			
0	777 (46.1)	1163 (51.5)	1940 (49.2)
1-4	807 (47.9)	963 (42.6)	1770 (44.9)
≥5	101 (6.0)	132 (5.8)	233 (5.9)
Comorbidities			
0-3	507 (30.1)	1155 (51.1)	1662 (42.1)
4-7	868 (51.5)	862 (38.2)	1730 (43.9)
≥8	310 (18.4)	241 (10.7)	551 (14.0)
Hospital location, rural	22 (1.3)	741 (32.8)	763 (19.3)
Hospital type, private	434 (25.8)	386 (17.1)	820 (20.8)
Booked admission	224 (13.3)	198 (8.8)	422 (10.7)

All figures shown as numbers (percentages) except where indicated. *ICD*, International Classification of Diseases; *MI*, myocardial infarction; *UA*, unstable angina; *CHD*, coronary heart disease.

### Prevalence of diabetes in CHD patients

In the ICD-9 sample, there was a small but significant difference between diabetes prevalence from medical records (22.5%) and index administrative data (21.5%, p < 0.0001) (Table 2). The difference was also significant (p < 0.0001) in the ICD-10 sample (34.9% from medical records compared with 29.7% from the administrative data), but prevalence increased to 34.2% using all previous hospital admissions to 15 years. There was a similar pattern in the Indigenous sample, although absolute prevalence levels were higher in this patient group.

**Table 2:** Prevalence of diabetes in coronary heart disease patients from medical records and hospital discharge data, stratified by lookback period

	**Whole sample**				Indigenous people^*^	
	**ICD-9, n (%)**		**ICD-10, n (%)**		**ICD-10, n (%)**	
	**(n = 1685)**		**(n = 2258)**		**(n = 525)**	
**Lookback period**	**Medical records**	**Hospital discharge data**	**Medical records**	**Hospital discharge data**	**Medical records**	**Hospital discharge data**
Index admission	380 (22.5)	363 (21.5) ^‡^	789 (34.9)	670 (29.7) ^‡^	295 (56.2)	256 (48.8) ^‡^
1 year		373 (22.1)		721 (31.9) ^‡^		274 (52.2) ^‡^
2 years		377 (22.4)		737 (32.6) ^‡^		280 (53.3) ^‡^
5 years		381 (22.6)		765 (33.9) ^‡^		290 (55.2) ^‡^
10 years		383 (22.7)		772 (34.2) ^‡^		294 (56.0)
15 years		385 (22.8)^†^		773 (34.2) ^‡^		294 (56.0)

*Indigenous patient sample from the ICD-10 sample only.

P-values are comparing prevalence from medical records with hospital discharge data, separately for ICD-9, ICD-10, and Indigenous sample. †p < 0.05; ‡p < 0.0001.

### Concordance measures

Observed agreement was high and Kappa very good in both samples and across all lookback periods for the recording of diabetes in the two data sources (Table 3). Sensitivity was significantly lower in the ICD-10 compared with ICD-9 sample from the index admission (81.5% versus 91.1%, p < 0.0001), but improved to 89.6% using 10 years of hospitalization history. NPV was significantly higher in the ICD-9 than ICD-10 sample (p < 0.0001 for all lookback periods), although the difference diminished with increasing lookback. Specificity was high in both samples, with a small decrease as lookback period increased. PPV declined with use of an increased lookback period, but there was no statistical difference between the two time periods. Diabetes status was underestimated by 15% from the hospital discharge index admission in the ICD-10 sample, which reduced to 2.2% using a 10-year lookback period (Table 3).

**Table 3:** Concordance measures for the recording of diabetes in hospital discharge data compared with medical records, in the sample of coronary heart disease patients

	**Observed agreement,%**		**Kappa,%**		**Sensitivity,%**		**Specificity,%**		**Positive predictive value,%**		**Negative predictive value,%**		Underestimation (−) / overestimation (+)^*^	
**Lookback period**	**ICD-9**	**ICD-10**	**ICD-9**	**ICD-10**	**ICD-9**	**ICD-10**	**ICD-9**	**ICD-10**	**ICD-9**	**ICD-10**	**ICD-9**	**ICD-10**	**ICD-9**	**ICD-10**
Index admission	97.0	92.3	91.2	82.5	91.1	81.5^‡^	98.7	98.2	95.3	96.0	97.4	90.8^‡^	−4.4	−15.1^†^
1 year	96.6	93.4	90.2	85.3	91.6	86.3^†^	98.1	97.3	93.3	94.4	97.6	93.0^‡^	−1.8	−8.6^†^
2 years	96.6	93.4	90.3	85.4	92.1	87.3^†^	97.9	96.7	92.8	93.5	97.8	93.4^‡^	−0.7	−6.6^†^
5 years	96.5	93.6	90.0	85.9	92.4	89.3	97.7	95.9^†^	92.1	92.2	97.8	94.4^‡^	+0.3	−3.1^†^
10 years	96.5	93.5	90.0	85.6	92.6	89.6	97.6	95.6^†^	91.9	91.6	97.8	94.5^‡^	+0.8	−2.2^†^
15 years	96.4	93.4	89.7	85.5	92.6	89.6	97.5	95.5^†^	91.4	91.5	97.8	94.5^‡^	+1.3	−2.1^†^

* Calculated from (Sensitivity/PPV – 1) x 100. Negative values represent the percentage underestimation and positive values the percentage overestimation of diabetes recording in hospital discharge data compared with medical records.

P-values are from comparison of ICD-9 and ICD-10 for each lookback period. †p < 0.05. ‡p < 0.0001.

*ICD*, International Classification of Diseases.

Because of the oversampling of Indigenous patients in Study 2, the ICD-10 sample was stratified to compare concordance for the ICD-10 patients from Study 1 only with the total ICD-10 sample (Study 1 plus Study 2) (see Additional file 1). There was little difference between the restricted and full sample for all concordance measures, with a similar pattern of increasing sensitivity and a small drop in PPV with increasing lookback period.

### Concordance measures in Indigenous patients

Sensitivity was higher in Indigenous compared with non-Indigenous people for every lookback period, although the difference was only significant for lookback periods of two years or more (Table 4). Maximal sensitivity was achieved with 10 years lookback in Indigenous patients (93.6%). Specificity was lower in Indigenous than non-Indigenous patients, with an increasing differential with increasing lookback period (p < 0.05). Diabetes status was underestimated from the hospital discharge index admission in Indigenous people by 13.3% reducing to 0.3% using a 10-year lookback period.

**Table 4:** Concordance measures for the recording of diabetes in hospital discharge data compared with medical records in Indigenous (n = 525) and non-Indigenous (n = 1733) coronary heart disease patients

**Lookback period**		**Observed Agreement,%**	**Kappa,%**	**Sensitivity,%**	**Specificity,%**	**Positive predictive value,%**	**Negative predictive value,%**	Underestimation (−) /overestimation (+) (%)^*^
Index admission	Indigenous	90.3	80.6	84.7	97.4	97.7	83.3^‡^	−13.3
	Non-Indigenous	93.0	81.4	79.6	98.3	94.9	92.3	−16.1
1 year	Indigenous	91.4	82.8	88.8	94.8^†^	95.6	86.8^‡^	−7.1
	Non-Indigenous	94.1	85.0	84.8	97.8	93.7	94.2	−9.5
2 years	Indigenous	92.2	84.2	90.5^†^	94.3^†^	95.4	88.6^‡^	−5.1
	Non-Indigenous	93.8	84.5	85.4	97.2	92.3	94.4	−7.5
5 years	Indigenous	92.9	85.7	92.9^†^	93.0^†^	94.5	91.1^†^	−1.7
	Non-Indigenous	93.8	84.7	87.2	96.4	90.7	95.0	−3.9
10 years	Indigenous	92.9	85.7	93.6^†^	92.2^†^	93.9	91.8	−0.3
	Non-Indigenous	93.6	84.3	87.2	96.2	90.2	95.0	−3.3
15 years	Indigenous	92.9	85.7	93.6^†^	92.2^†^	93.9	91.8	−0.3
	Non-Indigenous	93.6	84.1	87.2	96.1	90.0	95.0	−3.1

Concordance measures calculated using the ICD-10 sample only.

*Calculated from (Sensitivity/PPV – 1) x 100. Negative values represent the percentage underestimation and positive values the percentage overestimation of diabetes recording in hospital discharge data compared with medical records.

P-values are from comparisons between Indigenous and non-Indigenous patients for each lookback period. †p < 0.05, ‡p < 0.0001.

### False negatives and false positives

Significant univariable predictors in both periods for false negatives were elective admission, in-hospital death, and non-acute CHD principal diagnosis (Table 5). These remained significant after multivariable adjustment in both periods. A lower number of comorbidites (0–3) were associated with higher odds of a false negative in the ICD-10 but not the ICD-9 period. The level of false positives was low in both the ICD-9 (n = 22, 1.3%) and ICD-10 (n = 40, 1.8%) samples, with no significant univariable association with any of the variables tested, and therefore no multivariable analyses were undertaken.

**Table 5:** Characteristics associated with false negatives in administrative data on index admission

	**ICD-9**		**ICD-10**	
**Variable**	False negatives, n (%)^* ^**(n = 34)**	Multivariable model, odds ratio (95% CI)^*^	False negatives, n (%)^* ^**n = 146**	Multivariable model, odds ratio (95% CI)^*^
Admission Type				
Booked	12 (25.5)^†^	3.35 (1.36, 8.28)	22 (29.8) ^†^	1.96 (1.12, 3.44)
Emergency	22 (6.6)	-	124 (17.3)	-
In-hospital death				
Yes	8 (19.5)^†^	2.61 (0.97, 7.02)	15 (34.9) ^†^	3.70 (1.74, 7.85)
No	26 (7.7)	-	131 (17.6)	-
Length of stay (days)				
1-2	4 (7.3)^†^	0.47 (0.14, 1.56)	57 (27.3)^†^	1.66 (0.99, 2.77)
3-5	11 (5.3)	0.50 (0.21, 1.19)	62 (14.9)	0.91 (0.55, 1.49)
≥6	19 (16.4)	-	27 (16.6)	-
Principal diagnosis				
MI	16 (8.3)^‡^	0.29 (0.09, 0.86)	61 (16.4) ^†^	0.32 (0.16, 0.61)
UA	9 (5.6)	0.24 (0.07, 0.76)	66 (18.0)	0.34 (0.18, 0.66)
Other CHD	9 (33.3)	-	19 (36.5)	-
Comorbidities				
0-3	4 (5.3)	-	71 (23.4)^‡^	2.41 (1.20, 4.81)
4-7	15 (7.5)		58 (16.5)	1.78 (0.92, 3.45)
≥8	15 (14.3)		17 (12.7)	-

*Percentage is the proportion of false negatives within each category for each variable (FN / FN + TP in each category). Multivariable models are adjusted for all variables which are significant univariable predictors of false negatives in each period, and all are included in the models as categorical variables. Comorbidities was not included in the multivariable model for the ICD-9 sample. †p < 0.05, ‡p < 0.0001.

*ICD*, International Classification of Diseases; *CI*, confidence interval; *MI*, myocardial infarction; *UA*, unstable angina; *CHD*, coronary heart disease; *FN*, false negative; *TP*, true positive.

## Discussion

This study found that identification of diabetes status from linked administrative health data using the index CHD admission underestimated the prevalence of diabetes in CHD patients to a greater degree in the ICD-10 period, with a correspondingly lower sensitivity and NPV. Sensitivity improved from 81.5% to 89.6% using a 10 year lookback in the ICD-10 period, with marginal improvement in any measures with a longer lookback period out to 15 years. Sensitivity was higher and specificity lower for Indigenous compared with non-Indigenous CHD patients at the index admission and with an increasing lookback period. In-hospital death, elective admission type, and a non-acute CHD principal diagnosis were significantly associated with false negatives on the index CHD admission in both periods. The level of false positives was low in both periods.

Our results highlight the potential for changes in the accuracy of administrative data over time. Although specificity was high in both periods, sensitivity was significantly lower in the ICD-10 period. A study of myocardial infarction patients also found that following the change from ICD-9 to ICD-10, sensitivity reduced from 80% to 66% for diabetes with complications using hospitalization data [20]. In contrast, Chen et al. [21] found no impact of this change on the validity of diabetes and other comorbidities, possibly because of the use of multiple data sources (hospitalization and physician claims data). Our results suggest that although diabetes is reasonably accurately recorded in administrative data compared with other comorbidities [6,22-24], use of data from the index admission only would attenuate likely upwards trends in diabetes prevalence in this population of CHD patients because of the lower sensitivity and NPV in the more recent ICD-10 period. Use of prior hospitalization history would reduce this difference. For example, a 10 year lookback period would increase sensitivity from 81.5% to 89.6% and NPV from 90.8% to 94.5%, with little loss of specificity and still maintaining a high PPV (91.9%) in the ICD-10 sample, and provide similar levels of concordance to that of the ICD-9 sample.

Published comparisons for the accuracy of recording of diabetes in administrative data for Indigenous people are limited. A Canadian study found that sensitivity was higher (91.1% versus 86%) and specificity lower (92.8% versus 97%) for identifying prevalent diabetes cases in the Aboriginal compared with non-Aboriginal population [25]. Our results are consistent with this pattern. The likelihood of diabetes being recorded at index admission may be higher in Indigenous patients because diabetes is more actively diagnosed and treated during hospitalization due to the known high burden in this population. The significantly lower NPV in Indigenous people at index admission may result from the higher prevalence of diabetes in this group. However, despite an increased risk of CHD recurrence in Indigenous people [26], similar length of lookback periods (five to 10 years) optimized concordance measures between Indigenous and non-Indigenous people. Because the use of hospitalization history draws on all hospital admissions, not just those for CHD, this potentially reflects the higher hospitalization rates in all diabetics compared with the general population [27].

Whilst hospital morbidity data may underestimate population-level diabetes prevalence [28,29], our results demonstrate that use of a lookback period can provide an accurate measure of diabetes prevalence in a defined population such as hospitalized CHD patients. Comorbidities used in the Charlson Comorbidity Index identified from the index hospitalization are underestimated by 46% during the ICD-9 period [22], although there is some evidence that the use of the index admission only provides optimal model discrimination in mortality outcome studies [24,30]. Use of additional data sources such as claims data, where available, to identify diabetic status in this sample of patients may reduce the need for longer lookback periods. However, our results suggest that ICD-9 administrative data are reasonably accurate for identifying diabetic and non-diabetic cases but that the index admission alone may not correctly identify prevalent diabetic patients in the ICD-10 era, which is important information for jurisdictions where multiple data sources are not available.

The period differences shown in this study may relate to changes in coding practices. In many administrative health databases, conditions secondary to the principal reasons for admission to hospital are only coded if actively treated or investigated during the hospital stay [1]. However, during the 1990s in Australia, diabetes was required to be coded irrespective of documented intervention [1] which would contribute to the high levels of concordance between the two data sources in the ICD-9 period. Coding standards implemented with the introduction of ICD-10 reversed this requirement [7]. Further directives regarding coding of diabetic complications have apparently led to a marked increase in hospitalizations for complications of diabetes. This highlights the need to understand local coding directives and changes to standards which are relevant to the condition being investigated. Our results show that despite the incongruent effect of these coding changes, the use of specified lookback periods would allow for continuity in trends of diabetes prevalence in CHD patients.

In contrast to other studies, we found no significant association of increasing age or recent hospitalization with false negatives, and also no association of sex [31,32]. Differing and potentially changing impacts of age and sex mean that they are important variables for stratification in epidemiological studies of CHD trends [33] and our results show that such studies would not be biased across age and sex groupings. The only difference ascertained between time periods was an association of fewer comorbidities being recorded in the ICD-10 sample. This has important implications, as diabetes is more likely to be coded as a secondary than primary diagnosis [7]. It is unlikely that this finding is due to the number of available coding fields [21], as up to 21 diagnosis fields are available to researchers. Additional analysis of all CHD and CVD admissions in WA showed a small significant decrease in the number of comorbidities coded on admissions during the period of this study (data not shown), indicating a trend towards recording lower numbers of comorbidities during the more recent time period. A sensitivity analysis was undertaken where variables reaching significance at the p < 0.1 level in univariable analyses were included in the multivariable models. There were no differences in the significance levels of the existing variables in the models, which confirmed their significant association with false negatives as shown in the main analysis.

### Limitations

The generalizability of our results to other hospitalized conditions, particularly non-cardiac conditions, is uncertain because concordance has been specifically measured in a sample of CHD patients. However, within a restricted population hospitalized with CHD, administrative data appear to reliably detect diabetes status. Although we have used recording of diabetes status in the medical records as a reference standard, there are potential limitations in this data source. Patients with less severe diabetes who are treated with diet alone may be less likely to be recorded in medical records as diabetic. There is also the possibility of inaccurate transfer of comorbid conditions to the discharge summary, but review of the whole medical record including drug charts limits the impact of this on identifying diabetic patients. Our results may not be generalizable to patients aged over 80 years due to the age range selected in our sample. However, the lack of significant association between increasing age and under-recording of diabetes suggests that any difference in concordance measures in the very elderly may be small. The differences between the study samples could conceivably contribute to the differences in concordance measures demonstrated in our results, however, stratification by Indigenous status and by study source clearly demonstrate a similar pattern as seen in the main results. We were unable to demonstrate whether concordance measures have remained consistent since the latter period of our study.

## Conclusion

This study has identified a temporal difference in concordance between medical records and administrative health data for the identification of diabetes in CHD patients. In linked administrative data, using up to ten years of hospitalization history to identify diabetes status reduces the temporal difference, improving concordance levels in the later ICD-10 period to those of the ICD-9 period. The use of unlinked administrative data to identify diabetes status would still provide reasonably high levels of accuracy however trends over time would be biased and prevalence of diabetes underestimated in the later period. Importantly, the level of concordance was as high in Indigenous as non-Indigenous patients in this setting, supporting the use of administrative data to identify diabetic status in this population group where diabetes and CHD impose a significant burden.

## Abbreviations

CHD: Coronary heart disease; ICD: International Classification of Diseases; WA: Western Australia; HMDC: Hospital Morbidity Data Collection; CVD: Cardiovascular disease; PPV: Positive predictive value; NPV: Negative predictive value.

## Competing interests

The authors declare that they have no competing interests.

## Authors’ contributions

LN, TB, MK and JH conceived and designed the study; LN carried out the data analysis; FS and JK assisted with interpretation of data from source studies; LN, TB, MK, JH, FS and JK contributed to interpretation of study results; LN wrote the manuscript, and all authors reviewed the draft manuscript and approved the final version.

## Authors’ information

Lee Nedkoff, BSc(Physiotherapy), MPH, Research Associate and Doctoral Scholar, Cardiovascular Research Group, School of Population Health, The University of Western Australia.

Matthew Knuiman, BSc(Hons), PhD, Winthrop Professor, Senior Biostatistician, Cardiovascular Research Group, School of Population Health, The University of Western Australia.

Joseph Hung, MBBS, FRACP, FACC, Winthrop Professor, Professor of Cardiology and Consultant Cardiologist, School of Medicine and Pharmacology, Sir Charles Gairdner Hospital Unit, The University of Western Australia.

Frank Sanfilippo, BSc, BPharm, PGradDipPharm, PhD, Research Associate Professor, Cardiovascular Research Group, School of Population Health, The University of Western Australia.

Judith Katzenellenbogen, BSc(OccTher), BSc(Epidemiol, Hons), MSc, PhD, Research Associate Professor, Combined Universities Centre for Rural Health, The University of Western Australia.

Tom Briffa, BPhysEd, MPhysEd, PhD, Research Associate Professor and Head, Cardiovascular Research Group, School of Population Health, The University of Western Australia.

## Pre-publication history

The pre-publication history for this paper can be accessed here:

http://www.biomedcentral.com/1471-2288/13/121/prepub

## Supplementary Material

Additional file 1 Concordance measures for the recording of diabetes in hospital discharge data with medical records, comparing a restricted ICD-10 sample (Study 1 only, n = 1099) and the whole ICD-10 sample (Study 1 plus 2, n = 2258), stratified by lookback period. Click here for file
