# Supplementary material for: Concordance between administrative health data and medical records for diabetes status in coronary heart disease patients: a retrospective linked data study
Source: BMC Med Res Methodol. 2013 Oct 1;13:121. doi: 10.1186/1471-2288-13-121 (PMC3849847; doi:10.1186/1471-2288-13-121)
Supplement: Additional file 1 — Concordance measures for the recording of diabetes in hospital discharge data with medical records, comparing a restricted ICD-10 sample (Study 1 only, n = 1099) and the whole ICD-10 sample (Study 1 plus 2, n = 2258), stratified by lookback period. [file 1471-2288-13-121-S1.docx]

Additional File 1. Concordance measures for the recording of diabetes in hospital discharge data with medical records, comparing a restricted ICD-10 sample (Study 1 only, n=1099) and the whole ICD-10 sample (Study 1 plus 2, n=2258), stratified by lookback period.

|  | ICD-10 Sample | | | | | | | | | | | |
| --- | --- | --- | --- | --- | --- | --- | --- | --- | --- | --- | --- | --- |
|  | Observed agreement, % | | Kappa,  % | | Sensitivity,  % | | Specificity,  % | | Positive Predictive Value, % | | Negative Predictive Value, % | |
| Lookback  period | Restricted sample | Full sample | Restricted sample | Full sample | Restricted sample | Full sample | Restricted sample | Full sample | Restricted sample | Full sample | Restricted sample | Full sample |
| Index  admission | 93.3 | 92.3 | 83.2 | 82.5 | 82.0 | 81.5 | 98.1 | 98.2 | 94.7 | 96.0 | 92.8 | 90.8 |
| 1 year | 94.4 | 93.4 | 86.4 | 85.3 | 87.2 | 86.3 | 97.5 | 97.3 | 93.7 | 94.4 | 94.7 | 93.0 |
| 2 years | 94.3 | 93.4 | 86.0 | 85.4 | 87.5 | 87.3 | 97.1 | 96.7 | 92.9 | 93.5 | 94.8 | 93.4 |
| 5 years | 94.4 | 93.6 | 86.4 | 85.9 | 89.6 | 89.3 | 96.4 | 95.9 | 91.3 | 92.2 | 95.6 | 94.4 |
| 10 years | 94.2 | 93.5 | 86.0 | 85.6 | 89.6 | 89.6 | 96.1 | 95.6 | 90.7 | 91.6 | 95.6 | 94.5 |
| 15 years | 94.1 | 93.4 | 85.8 | 85.5 | 89.6 | 89.6 | 96.0 | 95.5 | 90.4 | 91.5 | 95.6 | 94.5 |

ICD, International Classification of Diseases.
